# A Prospective, Multicenter, Randomized Phase II Study to Evaluate the Efficacy and Safety of Eculizumab in Patients with Guillain-Barré Syndrome (GBS): Protocol of Japanese Eculizumab Trial for GBS (JET-GBS)

**DOI:** 10.2196/resprot.6610

**Published:** 2016-11-07

**Authors:** Nobuko Yamaguchi, Sonoko Misawa, Yasunori Sato, Kengo Nagashima, Kanako Katayama, Yukari Sekiguchi, Yuta Iwai, Hiroshi Amino, Tomoki Suichi, Takanori Yokota, Yoichiro Nishida, Nobuo Kohara, Koichi Hirata, Kazutoshi Nishiyama, Ichiro Yabe, Ken-Ichi Kaida, Norihiro Suzuki, Hiroyuki Nodera, Shoji Tsuji, Haruki Koike, Jun-Ichi Kira, Hideki Hanaoka, Susumu Kusunoki, Satoshi Kuwabara, JET-GBS Group

**Affiliations:** ^1^Clinical Research CenterChiba University HospitalChibaJapan; ^2^Department of NeurologyChiba University Graduate School of MedicineChibaJapan; ^3^Department of Global Clinical ResearchChiba University Graduate School of MedicineChibaJapan; ^4^Department of Neurology and Neurological ScienceTokyo Medical and Dental UniversityTokyoJapan; ^5^Department of NeurologyKobe City Medical Centre General HospitalKobeJapan; ^6^Department of NeurologyDokkyo Medical UniversityTochigiJapan; ^7^Department of NeurologyKitasato University School of MedicineKanagawaJapan; ^8^Department of NeurologyHokkaido University Graduate School of MedicineSapporoJapan; ^9^Division of NeurologyDepartment of Internal MedicineNational Defense Medical CollegeSaitamaJapan; ^10^Department of NeurologyKeio University School of MedicineTokyoJapan; ^11^Department of Clinical NeuroscienceGraduate School of Medical SciencesTokushima UniversityTokushimaJapan; ^12^Department of NeurologyThe University of Tokyo Graduate School of MedicineTokyoJapan; ^13^Department of NeurologyNagoya University Graduate School of MedicineNagoyaJapan; ^14^Department of Neurology, Neurological InstituteGraduate School of Medical SciencesKyushu UniversityFukuokaJapan; ^15^Department of NeurologyFaculty of MedicineKindai UniversityOsaka-SayamaJapan

**Keywords:** Guillain-Barré syndrome, eculizumab, complement activation, clinical trial, antiganglioside antibody

## Abstract

**Background:**

Guillain-Barré syndrome (GBS) is an immune-mediated neuropathy that causes acute flaccid paralysis. Immunoglobulin and plasma exchange are established treatments for GBS; however, a substantial number of patients, particularly those with severe disease, have poor recovery and residual deficits. Recent studies suggest that complement activation plays a pivotal role in GBS-associated axonal degeneration, and eculizumab is a humanized monoclonal antibody that specifically binds to complement component 5 and potently inhibits complement activation.

**Objective:**

This clinical trial aims to evaluate the efficacy and safety of eculizumab, a humanized monoclonal antibody directed against complement component 5, for treatment of GBS.

**Methods:**

The Japanese Eculizumab Trial for GBS (JET-GBS) is a prospective, multicenter, placebo-controlled, double-blind, randomized phase II study conducted at 13 tertiary neurology centers and is funded by the Japan Agency for Medical Research and Development. A total of 33 GBS patients unable to walk independently within 2 weeks from symptom onset (Hughes functional grade 3-5) were randomized at a 2:1 ratio to receive either intravenous eculizumab (900 mg/day) or placebo once weekly for 4 weeks, followed by 20 weeks of follow-up. The primary endpoint for efficacy is the proportion of patients who regain their ability to walk without aid at 4 weeks after the first dose of the study treatment, while primary safety outcomes are the incidence of adverse events and serious adverse events during the trial.

**Results:**

Enrollment for the trial began in August 2015. This trial is still ongoing. All participants have been enrolled, and follow-up will be completed in October 2016.

**Conclusions:**

This study is the first to investigate the efficacy and safety of eculizumab for GBS. In case of a positive result, we will plan a phase III trial to investigate this issue in a larger number of patients.

**ClinicalTrial:**

UMIN Clinical Trials Registry UMIN 000018171; https:/upload.umin.ac.jp/cgi-open-bin/ctr/ctr.cgi?function= brows&action=brows&type=summary&language=J&recptno=R000020978 (Archived by WebCite at http://www.webcitation.org/ 6lTiG8ltG). Clinical Trials.gov NCT02493725; https://clinicaltrials.gov/ct2/show/NCT02493725 (Archived by WebCite at http://www.webcitation.org/6lVJZXKSL)

## Introduction

Guillain-Barré syndrome (GBS) is the most common cause of acute tetraparalysis in developed countries [1,2]. GBS occurs in all age groups, and the annual incidence is reported to range from 0.81 to 1.89 per 100,000 persons [3]. Although the efficacies of intravenous immunoglobulin (IVIg) and plasma exchange have been demonstrated in randomized controlled studies [4,5], GBS still carries nonnegligible mortality and morbidity [6]. Mortality within the first year is approximately 4% [6]. Recovery takes several months or years, and approximately 14% of GBS patients show severe persistent disability at one year after onset [6].

GBS is classified into two major subtypes: acute inflammatory demyelinating polyneuropathy (AIDP) and acute motor axonal neuropathy (AMAN). Over the past two decades, major advances have been made in understanding the pathophysiology of AMAN [7]. It is now established that AMAN is caused by molecular mimicry of human gangliosides by an antecedent pathogen and ensuing antiganglioside antibody-mediated attack on the axolemma. In an animal model induced by antiganglioside antibodies, complement activation contributed to disruption of the nodal and paranodal molecular architectures, leading to acute paralysis and residual deficits [7-9]. Furthermore, nafamostat mesilate, a synthetic serine protease inhibitor with anticomplement action, prevented nerve damage in a GBS rabbit model [10].

Eculizumab is a humanized monoclonal antibody that specifically binds to complement component 5 (C5) and suppresses its cleavage into active chemotaxis mediator C5a and attacks complex component C5b, thereby inhibiting membrane attack complex (MAC) formation [11,12]. Eculizumab has been approved for treatment of paroxysmal nocturnal hemoglobinuria and atypical hemolytic uremic syndrome [13,14], disorders wherein intravascular hemolysis is induced by complement activation. The efficacy of eculizumab for GBS has been shown in a mouse model mediated by antiganglioside antibody [15]. Eculizumab blocks the formation of MAC associated with terminal axonal and glial injury at the neuromuscular junction and prevents respiratory paralysis [15]. We, therefore, designed the Japanese Eculizumab Trial for Guillain-Barré syndrome (JET-GBS) study to investigate the efficacy and safety of eculizumab in GBS patients.

## Methods

### Trial Design Overview

**Figure 1:**
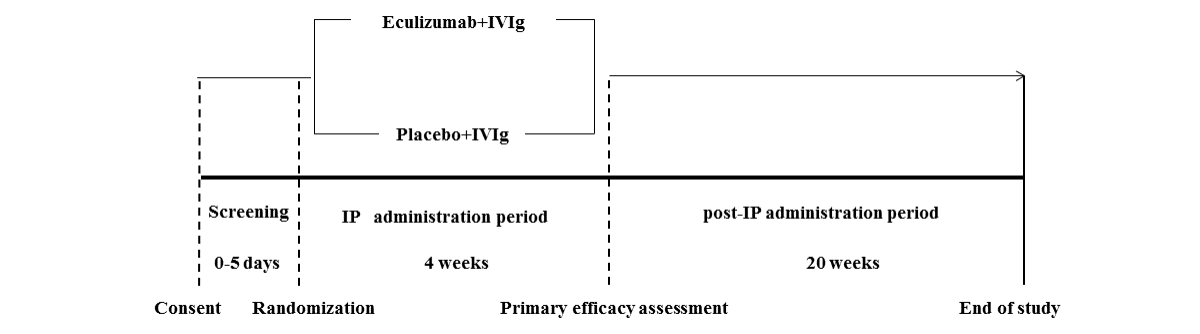
Schematic depiction of the trial design. The study is composed of 3 periods: the screening period, investigational product (IP) administration period (4 weeks), and post-IP period follow-up (20 weeks).

This is a prospective, multicenter, placebo-controlled, double-blind, randomized phase II trial in patients with GBS. A schematic depiction of the trial design is presented in Figure 1. The trial is composed of 3 periods: the screening period, investigation product (IP) administration period, and post-IP period. Screening is conducted within 5 days of randomization to assess eligibility and collect baseline data. Eligible patients were defined as those unable to walk independently (Hughes functional grade [FG] 3-5) within 2 weeks from the onset of weakness, because such patients have a higher risk of progression. They are randomized to receive either eculizumab or placebo at a 2:1 ratio. Eculizumab (900 mg/day) or matching placebo is administered intravenously once a week for a total of 4 doses (day 1, day 8, day 15, day 22) in conjunction with IVIg, the standard treatment for GBS (400 mg/kg daily for 5 consecutive days). IVIg can be readministered if patients show deterioration after initial improvement or stabilization (treatment-related fluctuation) following day 15. Patients are monitored periodically up to week 24. The primary endpoint for efficacy is the proportion of patients who reach FG 2 or lower (able to walk without aid) at day 29 (week 4). The primary endpoints for safety are the proportion and severity of adverse events (AEs) and serious adverse events (SAEs).

### Eligibility Criteria

GBS patients who meet all of the following inclusion criteria and none of the listed exclusion criteria are enrolled (see Textbox 1). Diagnosis of GBS is made with reference to the National Institute of Neurological Disorders and Stroke criteria [16].

**Selection criteria.**

Inclusion criteria:

- Patients 18 years or older at time of informed consent
- Presenting within 2 weeks from onset of weakness due to GBS
- Unable to walk unaided for 5 or more meters (Hughes FG 4-5; if symptoms are progressive, patients with FG 3 can be enrolled)
- Undergoing or being considered for and will start IVIg treatment (400 mg/kg daily for 5 days)
- The first dose of eculizumab administered within 2 weeks from onset of symptoms and before the last dose of IVIg
- If female, not pregnant. All patients must use effective contraception during the study and for up to 5 months following discontinuation of IP administration
- Hospitalization during the IP administration period
- Signed informed consent

Exclusion criteria:

- Patients who are or will be treated by plasmapheresis
- Pregnant or lactating woman
- Evident neuropathy other than GBS
- Patients who have received immunosuppressive treatment (eg, azathioprine, cyclosporine, tacrolimus, or more than 20 mg prednisolone daily) within 4 weeks prior to informed consent
- Patients with a severe concurrent disease such as malignancy, severe cardiovascular disease, severe chronic obstructive pulmonary disease, or tuberculosis
- Inability to comply with study procedures and the treatment regimen
- Patients who have received rituximab within 24 weeks prior to informed consent
- Unresolved *Neisseria meningitidis* infection or a history of meningococcal infection
- Active infection that is not treated appropriately
- Patients who cannot take antibiotics as *N meningitidis* prophylaxis due to allergies
- Known allergy to eculizumab
- Known hereditary complement deficiencies
- Use of other investigational products within 12 weeks prior to informed consent, or participation in any other clinical trial
- Any condition that in the opinion of the investigator or subinvestigator could increase the patient's risk by participating in the study or confound the outcome of the study
- Patients with a history of eculizumab treatment for GBS

### Interventions

#### Investigational Product (Eculizumab or Placebo)

Eculizumab (900 mg/day) or placebo is administered intravenously once a week for 4 weeks in patients who are being considered for, or already on IVIg treatment. The first dose of the IP is to be started before the last dose of IVIg. The patients are to be monitored by the medical staff for at least 1 hour following eculizumab/placebo infusion, because administration of eculizumab may result in infusion reactions. Administration is interrupted if severe infusion reactions occur.

#### Prophylactic Antibiotics

Inhibition of the terminal complement complexes predisposes patients to infections by encapsulated bacteria, especially *N meningitidis*. Therefore, all enrolled patients must receive antibiotic prophylaxis against *N meningitidis* for 8 weeks from the last dose of the IP.

### Outcomes

The efficacy and safety of eculizumab will be assessed by the following endpoints. The primary efficacy endpoint is the proportion of patients who reach FG 2 or lower (able to walk without aid) at week 4, and the safety endpoints are the proportion and severity of AEs/SAEs during the trial.

The secondary endpoints include the proportion of patients with improvement of 1 or more Hughes functional grades from baseline at each visit, proportion of patients who are able to walk unaided at each visit, duration required for improvement by at least 1 Hughes functional grade, proportion of FG 1 or 0 at week 24, change from peak FG at each visit, clinically relevant improvement in the Rasch-built Overall Disability Scale (R-ODS) score and the Overall Neuropathy Limitations Scale (ONLS) score at each visit, proportion of ventilatory support, duration of ventilatory support, occurrence of relapse, overall survival, changes in grip strength, manual muscle testing score, median and ulnar nerve conduction parameters, vital capacity respectively from baseline at each visit, and proportion of IVIg readministration.

This study is also investigating antiganglioside antibodies, concentration of serum eculizumab, and serum hemolytic activity as exploratory endpoints. The data of antiganglioside antibodies assay will be used to determine the subtype of GBS, and the results of eculizumab concentration and hemolytic activity will be used to obtain exploratory data on the efficacy of eculizumab.

### Study Visits and Assessments

The schedule for the study visits and assessments are summarized in Tables 1 and 2. Patients are assessed weekly for efficacy and safety during the 4-week IP administration period and on week 6, 8, 12, 16, and 24 during the 20-week post-IP period. Patients are requested to record their FG score independently using a diary until they show an improvement by one grade. If all 4 administrations of the IP cannot be completed due to AEs or other contingencies, patients remain in the trial and tests/assessments will be performed on the patients according to the trial schedule unless they meet the withdrawal criteria.

**Table 1:** Study visit and assessments during the screening and investigational product administration period.

Period		Screening	IP^a^ administration				
Trial visit		1	2	3	4	5	6
Trial weeks			W0	W1	W2	W3	W4
Trial days		D0	D1	D8	D15	D22	D29
Time window (days)		−5		±2	±2	±2	±7
Informed consent		X					
Randomization		X					
Demography and medical history		X					
IP administration			X	X	X	X	
Chemoprophylaxis^b^			X	X	X	X	X
**Clinical assessment**							
	FG^c^	X	X	X	X	X	X
	ONLS^d^		X	X	X	X	X
	MMT^e^ score		X	X	X	X	X
	Grip strength		X	X	X	X	X
	R-ODS^f^		X				X
**Physiological test**							
	Vital capacity	X					X
	Nerve conduction study	X					X
	12-lead ECG^g^	X		X			
Clinical lab test (blood test/urine test)		X		X	X	X	X
Antiganglioside antibody		X					
Eculizumab concentration/hemolytic activity			X	X			X
Pregnancy test (urine test)^h^		X					
Vital signs^i^		X	X	X	X	X	X
Concomitant drug/therapy review		X	X	X	X	X	X
AE assessment		X	X	X	X	X	X

^a^IP: investigational product.

^b^Chemoprophylaxis will begin at the start of eculizumab infusion on day1 and be continued till 8 weeks from the last dose of eculizumab.

^c^FG: Hughes functional grade.

^d^ONLS: overall neuropathy limitations score.

^e^MMT: manual muscle testing.

^f^R-ODS: Rasch-built overall disability scale.

^g^ECG: electrocardiography.

^h^Pregnancy tests must be performed on all women of child bearing potential at the specified time points and verified to have a negative result. Pregnancy test may also be performed at any visit at the investigator’s discretion.

^i^Vital signs: blood pressure, heart rate, body temperature.

**Table 2:** Study visit and assessments during the post–investigational product administration period.

Period		Post-IP^a^ administration (outpatient)					Early termination	Follow-up visit^b^
Trial visit		7	8	9	10	11		
Trial weeks		W6	W8	W12	W16	W24		
Trial days		D43	D57	D85	D113	D169		
Time window (days)		±7	±7	±7	±7	±7	+7	+7
Informed consent							
Randomization							
Demography and medical history							
IP administration							
Chemoprophylaxis^c^		X	X					
**Clinical assessment**							
	FG^d^	X	X	X	X	X	X	
	ONLS^e^	X	X	X	X	X	X	
	MMT^f^ score	X	X	X	X	X	X	
	Grip strength	X	X	X	X	X	X	
	R-ODS^g^		X	X	X	X	X	
**Physiological test**							
	Vital capacity					X	X	
	Nerve conduction study			X		X	X	
	12-lead ECG^h^					X	X	
Clinical lab test (blood test/urine test)		X	X	X	X	X	X	X
Antiganglioside antibody							
Eculizumab concentration/hemolytic activity		X						
Pregnancy test (urine test)^i^						X	X	X
Vital signs^j^		X	X	X	X	X	X	X
Concomitant drug/therapy review		X	X	X	X	X	X	X
AE assessment		X	X	X	X	X	X	X

^a^IP: investigational product.

^b^If a patient withdraws from the trial within 8 weeks after the last IP administration, a final follow-up visit will be performed at 8 weeks after the last IP administration for safety assessment.

^c^Chemoprophylaxis will begin at the start of eculizumab infusion on day1 and be continued till 8 weeks from the last dose of eculizumab.

^d^FG: Hughes functional grade.

^e^ONLS: overall neuropathy limitations score.

^f^MMT: manual muscle testing.

^g^R-ODS: Rasch-built overall disability scale.

^h^ECG: electrocardiography.

^i^Pregnancy tests must be performed on all women of child bearing potential at the specified time points and verified to have a negative result. Pregnancy test may also be performed at any visit at the investigator’s discretion.

^j^Vital signs: blood pressure, heart rate, body temperature.

### Data Management, Monitoring and Auditing

Study data is recorded in the electronic case report form (eCRF) of Medidata Rave, which was configured for this study by the data management division at Chiba University Hospital Clinical Research Centre. The eCRF data maintain anonymity and identify participating patients by their assigned identification codes. Independent monitors ensure that the study is properly conducted at each site in accordance with the protocol and good clinical practice and verify that the contents of case reports and other reports are current and accurate. The monitors and data managers assure the quality of the data at each stage of handling. Auditors who are independent from all divisions conducting the trial perform audits of the clinical trial sites and verify appropriate quality assurance.

### Reporting for Adverse Events and Serious Adverse Events

All AEs are recorded on the AE page of the eCRF. The investigator at each site endeavors to obtain the outcome and severity of all AEs. AEs are reported from the time of informed consent to the last trial visit (week 24); however, SAEs with a causal relationship to the IP are monitored until resolution if possible. All SAEs are reported to all investigators and assessed through a Web-based AE reporting system that has been developed by the Japan Medical Association, Centre for Clinical Trials. SAEs that have not been reported previously are reported to the Pharmaceuticals and Medical Devices Agency.

### Sample Size Calculation

The sample size was based on the results from our previous study [17] and our historical database of GBS patients. For the primary endpoint of efficacy, we estimated that the threshold value for Hughes FG 2 or lower at week 4 would be 50% and the expected value 80%. A sample size of 20 patients in the eculizumab group is required to achieve statistical significance level of 5% (1-sided) at 80% power. To allow for a 10% dropout rate, total sample size in the eculizumab group is 22. Additionally, 11 patients will be randomly assigned to the placebo control to collect efficacy and safety data. The total sample size was set at 33.

### Allocation

Eligible patients who provided informed consent are registered for study enrollment and are randomized to the eculizumab or placebo arm at a 2:1 ratio via a Web-based system developed by ADJUST Co Ltd. Allocation is performed using the minimization method with biased coin assignment [18,19] balancing for FG score (FG=3 or FG≥4) and age (younger than 60 years or 60 years and older).

### Blinding

Each vial of IP contains 300 mg of eculizumab or placebo. The placebo has an identical external appearance to that of eculizumab. All participating patients, investigators, study coordinators, data managers, and outcome assessors remain blinded to group assignment during the trial. In order to maintain blinding, measurements of serum 50% hemolytic complement activity are prohibited during the trial. The levels of antiganglioside antibodies, eculizumab concentrations, and hemolytic activities in serum are measured at a central laboratory and the results are masked to all patients and study personnel during the trial.

### Statistical Methods

Statistical analyses and reporting of this trial will be conducted in accordance with the Consolidated Standards of Reporting Trials statement guidelines with the primary analyses based on the intent-to-treat principle without imputing any missing observations. All efficacy analyses will be primarily based on the full analysis set, which includes all patients who have received at least one dose of the study treatment.

The primary endpoint for efficacy is the proportion of patients with FG≤2 at week 4. We hypothesized that response threshold at week 4 would 50% as H_0_ (null hypothesis), whereas the expected response rate would be 80% as H_1_ (alternative hypothesis). The exact 90% confidence interval will be calculated by the binominal distribution for the response rate of each treatment group. The confidence limits of the confidence interval will be assessed against the response threshold; however, in primary analysis, we will not perform a statistical testing for comparison of treatment groups.

For the patient characteristics, summary statistics were constructed using frequencies and proportions for categorical data and means and SDs for continuous variables. Patient characteristics were compared using a Fisher exact test for categorical outcomes and a Student *t* test or the Wilcoxon rank sum test for continuous variables, as appropriate. All comparisons were planned, and all *P* values are 2-sided. A value of *P*<.05 is considered statistically significant. Subgroup analysis will be performed for safety and efficacy depending on GBS subtype, baseline FG, and electrodiagnosis.

All statistical analyses are performed using SAS software version 9.4 (SAS Institute) and the R statistical program version 2.13 (The R Foundation). All statistical analyses are described in the statistical analysis plan, which will be fixed prior to database lock.

### Ethics and Dissemination

#### Research Ethics Approval and Protocol Amendments

The study protocol was approved by the institutional review board (IRB) at each site before the start of the trial. Substantial amendments of the study protocol must be approved by each IRB. The trial was registered at the University Hospital Medical Information Network clinical trials registry [20] and ClinicalTrials.gov [NCT02493725].

#### Informed Consent

All participants are fully informed about the nature, purpose, possible risks, and benefits of the study with both oral and written information approved by all IRBs. Participants are notified that they are free to withdraw from the study at any time. The participants are given the opportunity to ask questions and allowed time to consider the information provided before consent. The participant’s signed and dated informed consent is obtained before conducting any study procedures. A copy of the consent document and explanation document is given to the participant and the original copy of the consent document is retained at the clinical trial site.

## Results

The trial started enrollment in August 2015. All 34 participants have been enrolled according to plan. This trial is still ongoing. Follow-up will be completed in October 2016.

## Discussion

The JET-GBS is the first phase II trial to investigate the efficacy and safety of a monoclonal antibody specifically binding C5 for the treatment of GBS. Given that complement activation and MAC deposition is known to play a pivotal role in GBS-associated nerve degeneration [7,8,9], we speculate that eculizumab can effectively prevent nerve injury, thereby accelerating recovery from GBS.

The efficacies of plasma exchange and IVIg for GBS treatment were first established in the 1980s and 1990s [4,5], but subsequent clinical trials have failed to demonstrate beneficial effects of alternative therapeutic approaches such as interferon-β1a [21], brain-derived neurotrophic factor [22], and mycophenolate mofetil [22]. Thus, IVIg and plasma exchange are still the mainstays of GBS treatment, with IVIg preferred because of its greater convenience and availability. However, new treatment options are necessary because the prognosis is still far from satisfactory for many patients [6].

There are several limitations to this study. First, the number of enrolled patients is small as this trial was designed to provide proof of concept rather than to assess the superiority of different treatments. If this study demonstrates efficacy and safety for GBS, we will plan a phase III trial to confirm its effectiveness and safety in a larger number of patients. Second, the pathogenic mechanism targeted by eculizumab (complement activation) appears active only in conjunction with antiganglioside antibody related to AMAN [8,9], as no study has clearly demonstrated a contribution of complement activation to demyelination or axonal degeneration in AIDP. However, the finding of C3d and C5b-9 deposits along the outer surface of Schwann cells in an autopsy study of AIDP suggests that eculizumab may also be effective for AIDP [23]. In the current study, we are also planning to investigate whether GBS subtype (AMAN or AIDP) or the presence of antiganglioside antibodies can influence the efficacy of eculizumab. Third, 3% of Japanese have a mutation in C5 that renders them resistant to eculizumab therapy. It might substantially affect results of this study.

There has been no substantial progress in GBS treatment over the last two decades. However, recent progress in understanding GBS pathophysiology and the development of novel biological agents targeting these pathogenic pathways could enable alternative approaches to mitigate nerve injury in GBS. The present clinical trial based on a specific pathogenic mechanism of GBS may pave the way for improved GBS therapies.

## Abbreviations

AIDP: acute inflammatory demyelinating polyneuropathy
AE: adverse event
AMAN: acute motor axonal neuropathy
C5: complement component 5
eCRF: electronic case report form
FG: Hughes functional grade
GBS: Guillain-Barré syndrome
IP: investigational product
IRB: institutional review board
IVIg: intravenous immunoglobulin
JET-GBS: Japanese Eculizumab Trial for Guillain-Barré syndrome
MAC: membrane attack complex
ONLS: Overall Neuropathy Limitations Scale
R-ODS: Rasch-built Overall Disability Scale
SAE: serious adverse event
